# Lactobacilli Cell-Free Supernatants Modulate Inflammation and Oxidative Stress in Human Microglia via NRF2-SOD1 Signaling

**DOI:** 10.1007/s10571-024-01494-1

**Published:** 2024-09-17

**Authors:** Mariagiovanna Di Chiano, Maria Teresa Rocchetti, Giuseppe Spano, Pasquale Russo, Caterina Allegretta, Giampaolo Milior, Raffaella Maria Gadaleta, Fabio Sallustio, Paola Pontrelli, Loreto Gesualdo, Carlo Avolio, Daniela Fiocco, Anna Gallone

**Affiliations:** 1 Department of Translational Biomedicine and Neuroscience (DiBraiN), University of Bari Aldo Moro, Bari, Italy; 2 Department of Clinical and Experimental Medicine, University of Foggia, Foggia, Italy; 3 Department of Agriculture Food Natural Science Engineering (DAFNE), University of Foggia, Foggia, Italy; 4 Department of Food, Environmental and Nutritional Sciences, University of Milan, Milan, Italy; 5 Department of Medical and Surgical Sciences, University of Foggia, Foggia, Italy; 6 CIRB, Collège de France, Université PSL, CNRS, INSERM, 75005 Paris, France; 7 Department of Interdisciplinary Medicine (DIM), University of Bari Aldo Moro, Bari, Italy; 8 Department of Precision and Regenerative Medicine and Ionian Area (DiMePRe-J), University of Bari Aldo Moro, Bari, Italy; 9 Istituto Nazionale Biostrutture e Biosistemi INBB, Viale delle Medaglie d’Oro, Roma, Italy

**Keywords:** *Lactobacilli* CFS, LPS, Gut–brain axis, NRF2, Postbiotics, Cytokines

## Abstract

Microglia are macrophage cells residing in the brain, where they exert a key role in neuronal protection. Through the gut–brain axis, metabolites produced by gut commensal microbes can influence brain functions, including microglial activity. The nuclear factor erythroid 2-related factor 2 (NRF2) is a key regulator of the oxidative stress response in microglia, controlling the expression of cytoprotective genes. Lactobacilli-derived cell-free supernatants (CFSs) are postbiotics that have shown antioxidant and immunomodulatory effects in several in vitro and in vivo studies. This study aimed to explore the effects of lactobacilli CFSs on modulating microglial responses against oxidative stress and inflammation. HMC3 microglia were exposed to lipopolysaccaride (LPS), as an inflammatory trigger, before and after administration of CFSs from three human gut probiotic species. The NRF2 nuclear protein activation and the expression of NRF2-controlled antioxidant genes were investigated by immunoassay and quantitative RT-PCR, respectively. Furthermore, the level of pro- and anti-inflammatory cytokines was evaluated by immunoassay. All CFSs induced a significant increase of NRF2 nuclear activity in basal conditions and upon inflammation. The transcription of antioxidant genes, namely heme oxygenase 1, superoxide dismutase (SOD), glutathione-S transferase, glutathione peroxidase, and catalase also increased, especially after inflammatory stimulus. Besides, higher SOD1 activity was detected relative to inflamed microglia. In addition, CFSs pre-treatment of microglia attenuated pro-inflammatory TNF-α levels while increasing anti-inflammatory IL-10 levels. These findings confirmed that gut microorganisms’ metabolites can play a relevant role in adjuvating the microglia cellular response against neuroinflammation and oxidative stress, which are known to cause neurodegenerative diseases.

**Graphical Abstract:**

Gut-brain crosstalk: molecular point of view. Metabolites contained in the supernatant derived from Lactobacilli can cross the gut barrier and reach the central nervous system, where they are taken up by microglial cells. They induce the activation of the NRF2 pathway and the production of inflammatory mediators. This interaction attenuates two important events: oxidation (with high levels of NRF2) and inflammation (with high levels of IL-10 and low levels of TNF-α).

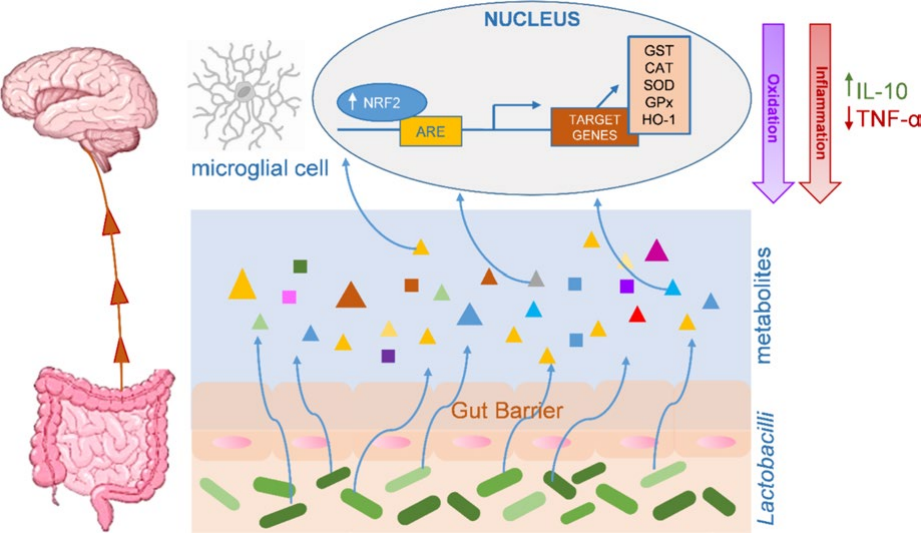

**Supplementary Information:**

The online version contains supplementary material available at 10.1007/s10571-024-01494-1.

## Introduction

Microglia are macrophages residing in the central nervous system (CNS), where they perform immune surveillance and control synaptic remodeling and neurogenesis (Ransohoff and El Khoury 2015; Abdel-Haq et al. 2019). As immunocompetent cells, microglia continuously survey the surrounding parenchyma and monitor signals arising from brain injuries or potential pathogens, hence being highly sensitive to both local and exogenous stimuli, including those coming from the gut (Abdel-Haq et al. 2019).

The gut microbiota comprises a vast and diverse microbial community that has a profound impact on human health. This complex ecosystem is considered a virtual organ that improves digestion of nutrients, benefits host metabolism, strengthens gut mucosal barrier, and modulates innate and adaptive immune responses (Evans et al. 2013). The gut microbiota influences also the physiology of organs and systems outside the gastro-intestinal tract. Indeed, through a complex network of interactions referred to as the gut–brain axis, it is known to modulate several facets of the CNS, including maturation and activation of microglia (Abdel-Haq et al. 2019; Carabotti et al. 2015). Altered composition of the gut microbiota, i.e., dysbiosis, may be detrimental to host health, being often associated to chronic diseases. For instances, intestinal dysbiosis have been observed in patients suffering from multiple sclerosis and other inflammatory neurological disorders (Abdel-Haq et al. 2019; Fettig and Osborne 2021; Dinan and Dinan 2022), whose pathogenesis is known to be associated to microglia dysfunctions. In fact, it is known that both in physiological and pathological conditions, microglial cells can be regulated by compounds, such as short-chain fatty acids (SCFAs) or lipopolysaccarides (LPS) originating from commensal intestinal bacteria (Erny et al. 2015). In response to these signals, microglia are able to activate their specific functions, bringing improvements or causing decompensations in pathological cases. For instance, the sudden activation of microglia by LPS was observed in a condition linked to synaptic disorders and long-term cognitive deficits (Jung et al. 2023). Once activated, microglia undergo morphological and functional switches, i.e., from a resting state, they can polarize toward a pro-inflammatory or anti-inflammatory phenotype (Cherry et al. 2014; Hu et al. 2015; Blandini 2013). Indeed, upon stimulation, in order to perform scavenging as well as tissue repair activities, microglia may acquire phagocytic functions, produce reactive oxygen species, secrete neurotrophic factors and produce a variety of inflammatory mediators (Blandini 2013).

One of the major regulators of the response to oxidative damage in microglia is the nuclear factor erythroid 2-related factor 2 (NRF2). Under physiological conditions, NRF2 is localized in the cytosol, where it binds to Kelch-like ECH-associated protein (KEAP1), which regulates its proteasome-mediated degradation (Ngo and Duennwald 2022). Following oxidative stress, NRF2 dissociates from KEAP1, escapes proteasomal degradation, and translocates into the nucleus, thus activating the transcription of a pool of genes encoding enzymes, such as heme oxygenase 1 (HO-1), superoxide dismutase (SOD), glutathione-S transferase (GST), glutathione peroxidase (GPx), and catalase (CAT) (Zhang et al. 2004; Yamamoto et al. 2008; Zhang and Hannink 2003), which are responsible for various detoxification and antioxidant defense processes. Besides, NRF2 plays an important role in regulating the anti-inflammatory response in microglia (Huang et al. 2015; Li et al. 2015).

Psychobiotics are probiotics that confer health benefits on the activities of the CNS, including cognitive functions (Bermúdez-Humarán et al. 2019). The communication axis between the gut and the brain involves the enteric nervous system (ENS), which, through the production of various neurotransmitters, such as acetylcholine and serotonin, mediates possible environmental changes, contributing to immune defenses (Sarkar et al. 2016; O’malley et al. 2010). Among the most studied psychobiotics, it has been observed that lactobacilli and bifidobacteria can reduce the inflammatory state of some neurological disorders through the secretion of metabolites, such as SCFAs (Tankou et al. 2018; Kouchaki et al. 2017). Indeed, postbiotics, defined as non-viable probiotic cells, including their components and metabolites, have been shown to possess similar health-promoting characteristics to the probiotics from which they originate (Salminen et al. 2021; Aguilar-Toalá et al. 2021).

Although emerging studies have revealed the potential anti-inflammatory role of probiotic-derived cell-free culture supernatants (CFSs) (De Marco et al. 2018; Rocchetti et al. 2023; Frick et al. 2007; Ren et al. 2020; Bermudez-Brito et al. 2013; 2015), data on their anti-inflammatory and antioxidant properties on human brain and on microglial cells are missing. In the present study, we evaluated whether probiotic-derived metabolites modulate the responses of microglia. To this aim, HMC3 immortalized human microglial cells were treated with CFSs from three probiotic species which are known to colonize the human intestine. Then, levels of activated NRF2 antioxidant system, its target genes, and inflammatory markers were evaluated under conditions simulating a pro-inflammatory environment.

## Materials and Methods

### Reagents

Minimum essential medium (MEM), fetal bovine serum (FBS), and Dulbecco’s phosphate-buffered saline (DPBS) were from Corning (Manassas, VA, USA); trypsin–EDTA, penicillin, streptomycin, amphotericin B, and L-glutamine were from Euroclone (Carlsbad, CA, USA); 3-(4,5-dimethylthiazol-2-yl)-2,5-diphenyl tetrazoliumbromide (MTT), dimethyl sulfoxide (DMSO), and lipopolysaccharides (LPS) from *Escherichia coli* O127:B8 were purchased from Sigma-Aldrich (St Louis, MO,USA); de Man-Rogosa-Sharpe (MRS) broth was from Oxoid (Basingstoke, UK).

### Bacterial Cultivation and Preparation of Cell-Free Supernatants

The following bacterial strains were from the Spanish Culture Collection (Colección Espaňola de Cultivos Tipo, CECT, Paterna, Spain): *Limosilactobacillus reuteri* NCFB 2589 (CECT 925) (Lr 13), *Lacticaseibacillus rhamnosus* NCIMB 8010 (CECT 278) (Lrh 19), *Lactiplantibacillus plantarum* (CECT 8328) (Lp 10). Bacteria were cultivated in MRS medium at 37 °C. Their cell-free supernatants (CFSs) were obtained by centrifugation (5,000 × rpm, 10 min) and filtration (0.45 µm) of stationary phase cultures (with an estimated concentration of 2–5 × 10^8^ colony-forming units [CFU] per mL).

### Cell Culture

The Human Microglia Clone 3 (HMC3), a line derived from human embryonic microglial cells (Janabi et al. 1995), were cultured in MEM supplemented with 10% FBS, 100-U/mL penicillin/streptomycin, and 100-U/mL amphotericin B, at 37 °C in a humidified incubator, under 5% CO_2_.

### MTT Assay

Based on our earlier work (Rocchetti et al. 2023), HMC3 cells were treated with 5% or 10% (v/v) CFSs from *L. plantarum*, *L. reuteri,* and *L. rhamnosus* to evaluate their cytotoxicity. Cells were seeded (2 × 10^4^ / well) into a 96-well culture plate and cultured for 24 h (Rocchetti et al. 2023). After 24 h, the culture medium was removed and microglia cells were incubated with 0.5-mg/mL MTT for 4 h at 37 °C. Then, formazan crystals were dissolved with DMSO and the absorbance at 540 nm was immediately measured. CFS-untreated cells were used as positive control, defining 100% viability. Based on the toxicity revealed by the MTT test (table S1), a concentration of 5% (v/v) CFS, i.e., allowing a viability above or equal to 80%, was regarded as safe, and thus selected to treat the HMC3 cells (see below).

### Treatment of Microglia Cells with Bacterial CFSs

**Scheme 1:**
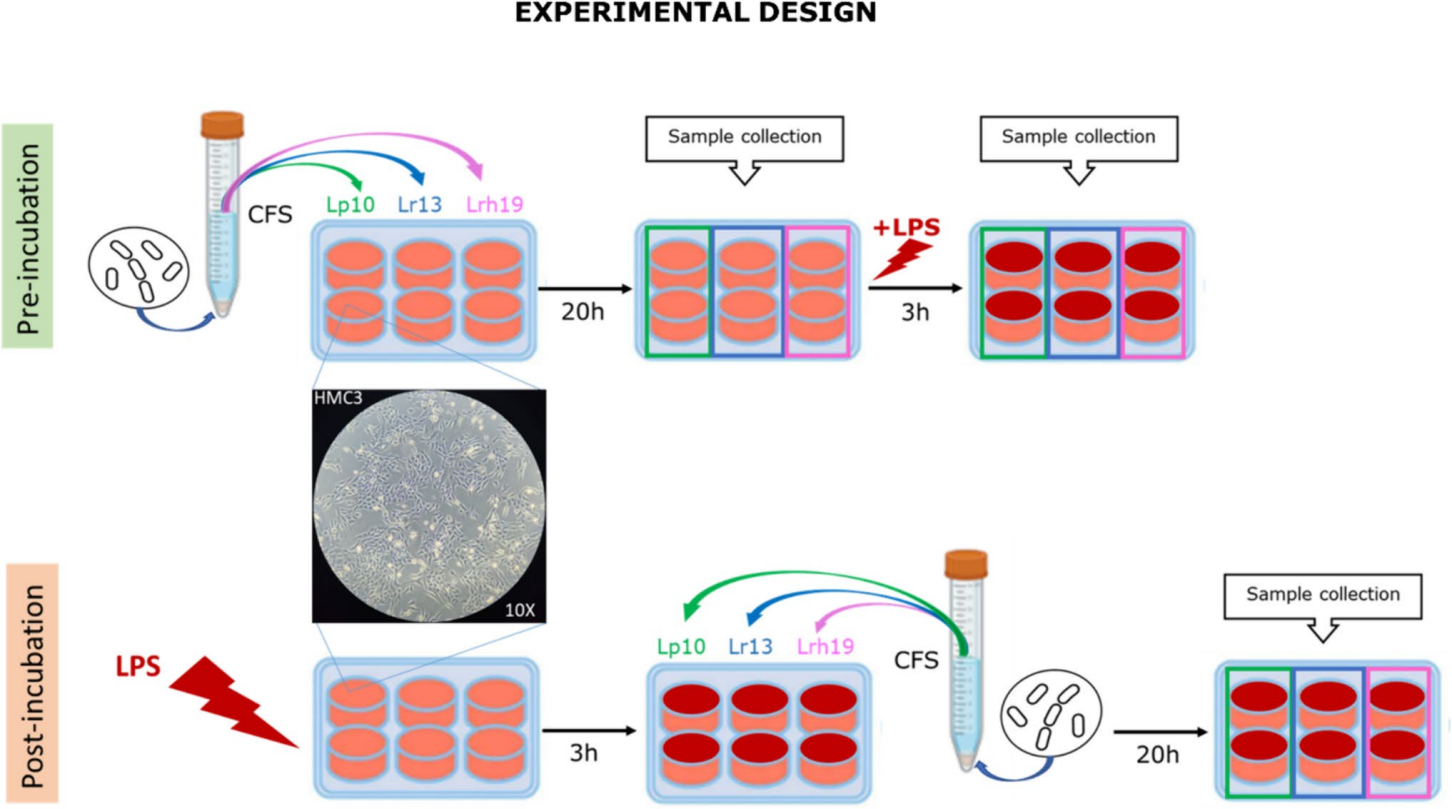
Experimental design of the treatment of human microglia cells (HMC3) with bacterial CFSs. Samples from different treatments were collected and processed for ELISA, transcriptional analyses, and enzymatic activity

HMC3 cells (3 × 10^5^ cells/mL) were seeded into a 6-well tissue culture plate and cultured at 37 °C until confluence was reached. Two conditions were set-up in order to evaluate the capacity of CFSs to modulate inflammation and redox status (Scheme 1). In the pre-incubation condition, HMC3 cells were pre-treated with CFSs 5% (v/v) for 20 h. Then, the medium was removed and cells were stimulated for additional 3 h with 1 µg/mL LPS. In the post-incubation condition, HMC3 were stimulated first with LPS (1 µg/mL) for 3 h. Then, the culture medium was removed and cells were incubated with CFSs for additional 20 h. The negative and positive controls were represented by CFS-untreated HMC3 not incubated and incubated with LPS, respectively. After each treatment, cells and /or their conditioned medium were processed for subsequent analysis.

### Transcriptional Analysis of Genes Involved in Oxidative Stress

Total RNA was isolated from treated and control HMC3 cells using the RNeasy Kit (Zymo Research, Orange, CA, USA). Purity and concentration of the RNA samples were determined by NanoDrop™ instrument (Thermo Fisher Scientific, Waltham, MA, USA). cDNA was synthesized from 500 ng of total RNA, using the iScript cDNA Synthesis Kit (Applied Biosystems, Waltham, MA, USA). cDNAs were stored at − 80 °C until analysis. To analyze the transcriptional level of genes encoding superoxide dismutase (SOD), catalase (CAT), glutathione-S transferase (GST), glutathione peroxidase (GPx), heme oxygenase 1 (HO-1), and interleukin-1β (IL-1β), a quantitative RT-PCR was performed in a real-time instrument (Applied Biosystems, USA), using SsoAdvanced™ Universal SYBR Green Supermix (Bio-Rad Laboratories, Hercules, CA, USA). The oligonucleotide primers (Sigma-Aldrich, USA) derived from Origene™ Technologies (table S2) were used at a concentration of 0.25 µM. PCR were performed using the following temperature profile: initial denaturation 95 °C for 30 s and 45 cycles of 95 °C for 10 s, 60 °C for 60 s, and 72 °C for 30 s. The PCR specificity was determined through melting curve analysis. The housekeeping genes encoding β-actin and glyceraldehyde-3-phosphate dehydrogenase (GAPDH) were used to normalize the expression level of target genes using the ΔΔCt method.

### Quantification of HMC3-Secreted Cytokines and Nuclear Level of NRF2

To analyze the level of secreted IL-10, TNF-α and IL-8, supernatants from HMC3 cultures were collected and stored at -80 °C until further analysis. The concentration of secreted cytokines was determined by ELISA assay (codes A103966, A78896 and A1476, Antibodies, Cambridge, UK,) according to the manufacturer’s guidelines.

In order to measure NRF2 nuclear activity levels, pellets from microglia cell cultures were obtained by centrifugation (1,100 × rpm, 10 min), and total nuclear proteins were isolated using a nuclear extraction kit (ab113474-Abcam, Cambridge, UK), according to manufacturer’s instructions. Total protein concentration was measured using Pierce™BCA Protein Assay kit (Thermo Fisher Scientific), and the nuclear activation of NRF2 was determined by a colorimetric assay (ab207223-Abcam) according to the manufacturer’s guidelines. The NRF2 levels were normalized to those of the control HMC3 cells (O.D. ratio).

### SOD Activity

Cytoplasmic SOD1 and mitochondrial SOD2 activities were assessed using the SOD colorimetric Activity Kit (Arbor Assays, Ann Arbor, MI, USA) according to manufacturer’s guidelines.

### Statistical Analysis

Statistical analysis of experimental data was performed using multiple unpaired, non-parametric Mann–Whitney t-tests and ANOVA followed by Tukey’s post hoc test, to compare the averages of two or more groups, respectively. Variables means and standard deviations (SD) were calculated, for each experimental condition, from at least three independent biologic replicates. All calculations were performed using StatView software package SAS (v. 5.0). *p* < 0.05 was considered statistically significant.

## Results

### CFSs Increase NRF2 Nuclear Activity and Transcription of Antioxidant Genes

**Fig. 1:**
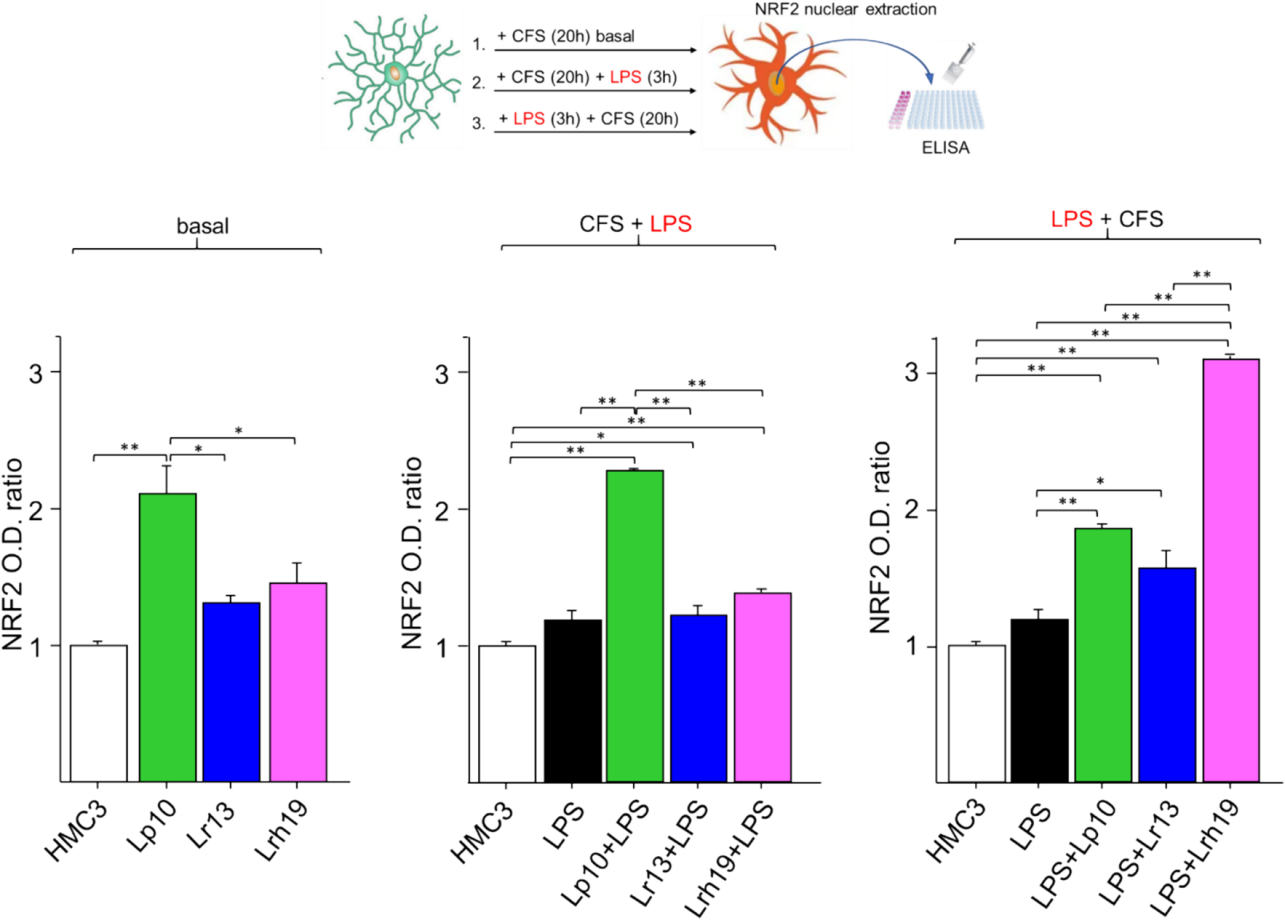
Nuclear activity of NRF2. NRF2 activity was assessed by an ELISA-based colorimetric assay on nuclear extracts from untreated microglia (open bar), LPS-treated cells (solid bars), and cells treated for 20 h with CFS from each bacterial strain (Lp10, Lr13, Lhr19) without (basal), before (CFS + LPS), or following (LPS + CFS) 3-h LPS stimulation. NRF2 activation was normalized to that detected in untreated control cells and expressed as optical density (O.D. 450 nm) ratio. The results represent the mean ± SD from three independent experiments. Statistically significant differences were determined by one-way ANOVA and Tukey’s post hoc test, *p < 0.05, **p < 0.01. CFSs were used at 5% (v/v) concentration; LPS was used at the concentration of 1 µg/mL

In order to evaluate the antioxidant properties of lactobacilli-derived metabolites, we monitored NRF2 nuclear activation in microglia incubated for 20 h with the CFSs from each of the investigated species (Fig. 1). CFSs from all tested lactobacilli tended to increase NRF2 level under basal conditions (i.e., in the absence of LPS stimulation) relative to untreated microglia, with Lp10 CFS inducing a statistically significant increase compared to untreated control cells (*p* < 0.01). Significantly increased NRF2 nuclear activity (*p* < 0.01) was also observed in microglia pre-treated with Lp10 CFS and then subjected to LPS treatment (CFS + LPS). When microglia were first treated with LPS and then with CFSs (LPS + CFS), the highest NRF2 level resulted from Lrh19 CFS treatment, although all tested CFSs significantly augmented NFR2 nuclear localization (*p* < 0.01) relative to untreated and inflamed HMC3 cells.

**Fig. 2:**
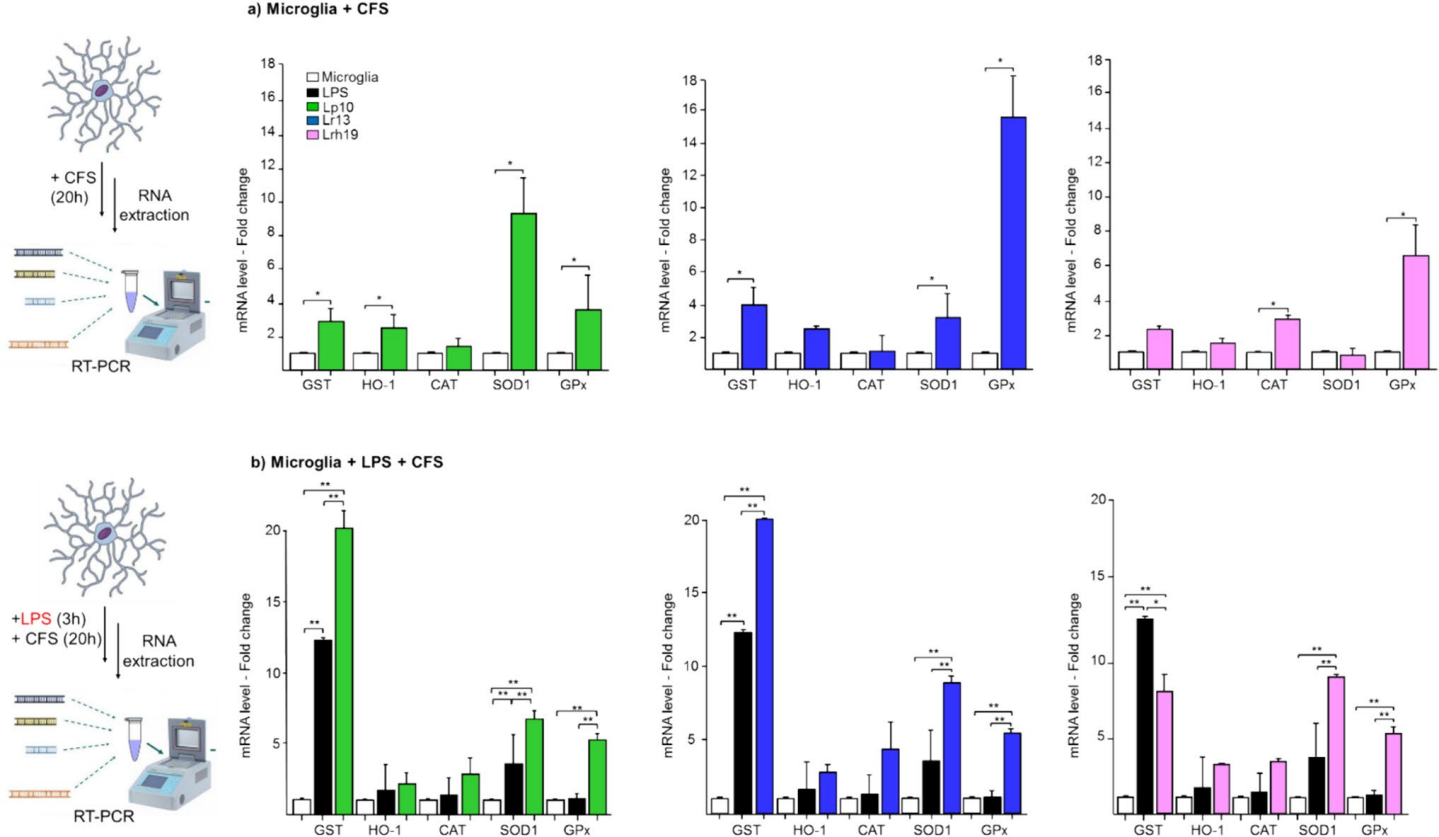
Transcriptional level of NRF2-controlled genes. **a** Relative transcriptional level in untreated microglia cells (white bars) and upon incubation for 20 h with CFS from Lp10 (green bars), Lr13 (blue bars), and Lrh19 (pink bars). **b** Relative transcriptional level in untreated microglia (white bars), in LPS-treated microglia (solid bars), and in LPS-treated microglia following 20-h incubation with CFS from each of the indicated *Lactobacilli* strains (same color code as in A). mRNA levels were determined by qRT-PCR and by normalizing to that of untreated control cells. Mean and SD from three independent experiments. Statistically significant differences were determined by Mann–Whitney *t* tests or by ANOVA followed by Tukey’s post hoc test, as appropriate. *p < 0.05, **p < 0.01. GST (glutathione-S transferase), HO-1 (heme oxygenase 1), CAT (catalase), SOD (superoxide dismutase), GPx (glutathione peroxidase). CFSs were used at 5% (v/v) concentration; LPS was used at the concentration of 1 µg/mL

In order to better ascertain the potential involvement of NRF2 as part of the effects of lactobacilli CFSs, the transcriptional level of genes directly controlled by NRF2 was analyzed (Fig. 2). In the absence of LPS treatment, CFSs from all three probiotic species significantly increased the expression of glutathione peroxidase (GPx) gene, while glutathione-S transferase (GST) transcription was significantly increased only by Lp10 and Lr13 (Fig. 2a). In particular, Lr13 CFS induced the highest expression of both genes. The superoxide dismutase (SOD1) gene mRNA level was significantly increased upon treatment with Lp10 and Lr13 CFSs, with Lp10 inducing its highest expression. Conversely, only Lrh19 induced a significant up-regulation of the catalase (CAT) gene (Fig. 2a). Incubation of LPS-inflamed microglia with CFS from all three probiotic species resulted in a significant beneficial modulation of most of the antioxidant genes (Fig. 2b). All CFSs induced a statistically significant increase of GPx and SOD1 expression (*p* < 0.01), both relative to untreated control and to LPS-stimulated cells (Fig. 2b). The expression of CAT and HO tended to be increased by all CFSs, albeit without statistical significance. GST gene was greatly induced by CFS from Lp10 and Lr13, with statistically relevant increase compared to both untreated and LPS-treated cells (*p* < 0.01). Compared to untreated control, LPS stimulation alone significantly upregulated (*p* < 0.01) only GST gene. The transcriptional levels of these genes were also analyzed in microglia pre-treated with CFS and then inflamed with LPS; however, they were found to be not significantly different from the control (data not shown).

### CFSs Enhance SOD Activity

**Fig. 3:**
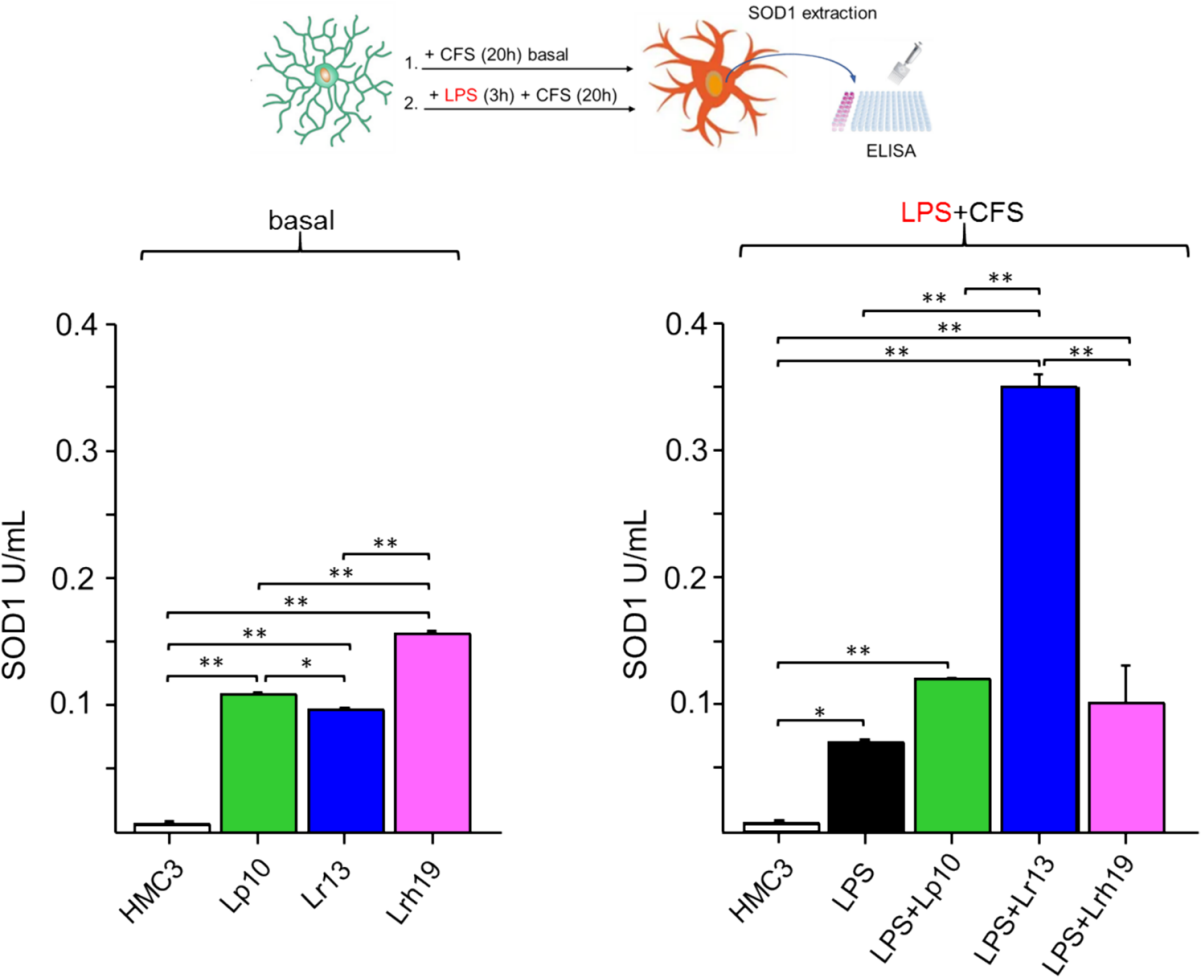
Activity of SOD1. SOD1 activity was assessed in untreated microglia (open bar), in LPS-treated cells (solid bar), and in cells treated for 20 h with 5% (v/v) CFS from each bacterial strain (Lp10, green bars; Lr13, blue bars; Lhr19, pink bars) in the presence of absence of 1 µg/mL LPS (LPS + CFS). Results expressed in U/mL represent the mean ± SD from three independent experiments. Statistically significant differences were assessed by one-way ANOVA followed by Tukey’s post hoc test. *p < 0.05; **p < 0.01

In an attempt to verify CFSs-dependent anti-oxidative effect on microglial cells, we measured the enzymatic activity of SOD1, whose gene is controlled by NRF2 and whose transcription was found to be significantly modulated by all CFSs in the post-incubation condition. SOD1 activity was determined in microglia cells treated for 20 h with CFSs (Fig. 3). In agreement with gene expression data, all CFSs significantly augmented SOD1 activity in the absence of LPS stimulation compared to untreated microglia. In particular, Lrh19 highly induced the activity of SOD1 in basal conditions, although it seemed not to modulate its gene expression (Fig. 2a). In line with gene expression analysis (Fig. 2b), following stimulation with LPS, incubation with CFSs, and particularly Lr13 CFS significantly raised SOD1 activity. Since ROS-scavenging activity is carried out also by SOD2, and such enzyme is known to be induced in activated microglia (Ishihara et al. 2015), we quantified its activity, finding it enhanced upon treatment with all CFSs, both in basal- and in LPS-treated cells, especially for Lr13 (Supplementary Fig. S1).

### CFSs Modulate the Level of Secreted Inflammatory Mediators

**Fig. 4:**
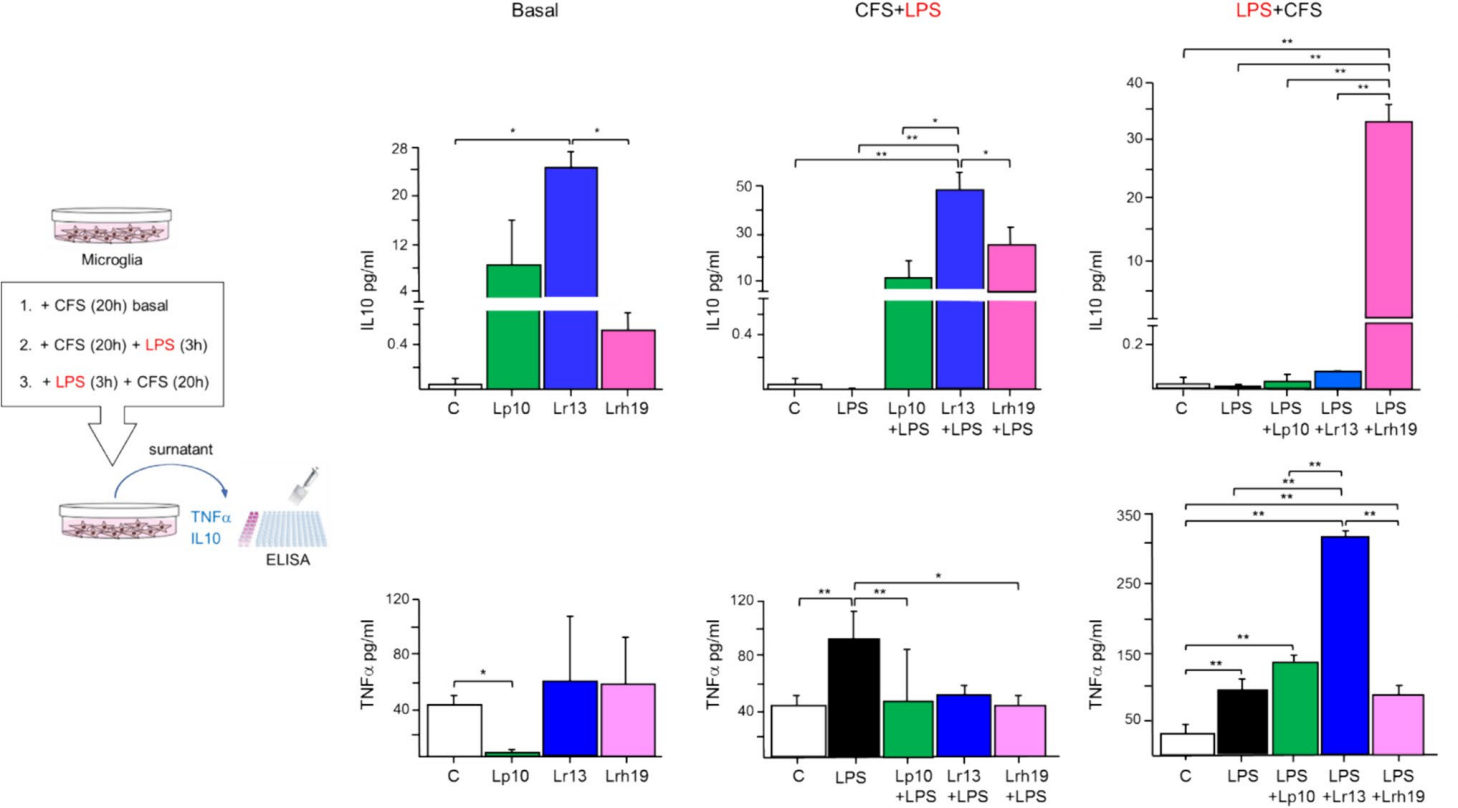
Level of secreted cytokines. The concentrations (pg/mL) of IL-10 (upper panel) and TNF-α (lower panel) were determined by ELISA in untreated microglia cells (control, white bars) and in microglia treated for 20 h with CFS from the different bacterial species (basal); in 3-h LPS-treated microglia (solid bars) and in 3-h LPS-treated microglia pre-incubated with CFS for 20 h (CFS + LPS); and in 3-h LPS-treated microglia (solid bars) and in 20-h CFS-incubated microglia following 3-h LPS stimulation (LPS + CFS). The results represent mean ± SD from three independent experiments; statistically significant differences were determined by one-way ANOVA followed by Tukey’s post hoc test. *p < 0.05; **p < 0.01. CFSs were used at 5% (v/v) concentration; LPS was used at the concentration of 1 µg/mL

In order to assess the inflammatory properties of CFSs, we monitored the secretion of TNF-α and IL-10 from microglia incubated with CFSs alone, incubated with CFSs and subsequently stimulated with LPS or treated with LPS and subsequently incubated with CFSs (Fig. 4).

In the absence of LPS, i.e., under basal condition, treatment with CFSs tended to increase the level of the anti-inflammatory cytokine IL-10 (Fig. 4). Such increase was statistically significant for Lr13 CFS, thus showing the best anti-inflammatory effect on basal conditions. The addition of LPS to CFSs-treated microglia (CFS + LPS) increased even more the level of secreted IL-10, compared to unstimulated and LPS-treated microglia, suggesting anti-inflammatory properties of all CFSs, more evident for Lr13. When microglia were pre-stimulated with LPS and then incubated with CFS (LPS + CFS), only Lrh 19 CFS determined significantly higher levels of secreted IL-10 (Fig. 4).

When evaluating the basal level of the pro-inflammatory molecule TNF-α, incubation with Lp10 CFS led to its significant reduction (*p* < 0.05), compared to control (Fig. 4). When a pro-inflammatory stimulus was added to cells pre-incubated with CFSs, all CFSs attenuated TNF-α production compared to LPS-activated microglia, although this effect was statistically significant only for CFS from two strains. On the contrary, the subsequent addition of CFSs to LPS-activated microglia exacerbated the pro-inflammatory response, increasing TNF-α secretion for Lp10 and significantly for Lr13 (Fig. 4).

When considering the expression of other pro-inflammatory cytokines, namely IL-1β mRNA level and IL-8 protein level (Supplementary Fig. S2), the capacity to down-regulate these signals under basal conditions was significant only for strain Lr13, while the preventive and post-incubation anti-inflammatory effect was confirmed for CFSs from all strains, although only in relation to secreted IL-8.

## Discussion

Microglia play homeostatic and reparative functions within the CNS; however, their defense reaction can be inadequate, putting the brain microenvironment at risk of neuroinflammation (Koutsilieri et al. 2002), which can lead to neurodegeneration (Zhang et al. 2023). The immune activity of microglia can be stimulated by a plethora of physiological and stress stimuli. Thanks to gut–brain interaction pathways, brain cells are exposed to microbial metabolites originating in the intestine. Several compounds deriving from the human gut microbiota are recognized as neuroprotective*,* because they exhibit anti-inflammatory and anti-oxidative effects in the context of different neurodegenerative diseases (Wang et al. 2022). CFSs contain a wide range of compounds secreted during bacterial growth: organic acids, fatty acids, esters, alcohols, phenolics, peptides, and specific secondary metabolites (Ramos et al. 2015; Mani López et al. 2022). As CFS are mixtures of diverse bioactive compounds, they exhibit various biologic activities. *Lactobacilli* CFSs were previously shown to modulate inflammatory and oxidative responses both in vitro (De Marco et al. 2018; Kwun et al. 2024; Dubey et al. 2021; Hao et al. 2023; Qadi et al. 2023; Chakamian et al. 2023) and in vivo (Dubey et al. 2021; Xu et al. 2022). The antioxidant effect of such type of postbiotics, specifically impacting NRF2 signaling and related downstream genes, has been reported in some recent studies (Karaca et al. 2022; Şirin 2023; Gholami et al. 2023; Zhang et al. 2022); however, data on human microglia are lacking.

To examine the anti-inflammatory and antioxidant properties of CFSs on human microglial cells, we adopted in vitro experimental conditions that could simulate neuroinflammation, i.e., we activated HMC3 cells with LPS, which are known to induce inflammation and oxidative stress in microglia (Hanisch 2002; Block and Hong 2005). Indeed, LPS act as immunogens inducing a microglial pro-inflammatory phenotype (Cherry et al. 2014; Hu et al. 2015; Block and Hong 2005). We focused on three species of lactobacilli, i.e., *L. plantarum, L. reuteri* and *L. rhamnosus*, since such probiotics can colonize the human gut and were previously shown to exert beneficial effects in animal models of neuroinflammation (Zolfaghari et al. 2021), cognitive (Xu et al. 2022), and neurological (Wu et al. 2022) dysfunctions. Moreover, these bacterial species possess the qualified presumption of safety (QPS) (EFSA BIOHAZ Panel 2024) and their therapeutic properties have been documented by several studies (Yadav et al. 2020), including clinical trials (https://clinicaltrials.gov/; https://www.who.int/ictrp/en/), also related to neurological disorders (Wiegers et al. 2022). We tested bacterial metabolites rather than live bacterial cells, indeed, compared to probiotics, postbiotics are safer, allow more stable formulations, and are tailorable for specific needs (Nataraj et al. 2020). Moreover, some tissues and organs, such as brain, are more likely to be influenced by microbial molecules rather than viable probiotic cells (Erny et al. 2015; Cosola et al. 2018).

In the present study, we analyzed the effects of CFSs on both naive and LPS-activated HMC3 cells. To quantify NRF2 activity, we employed a kit that assays its DNA-binding capacity: such method has been adopted in some recent papers studying NRF2 signaling and oxidative stress (Clementi et al. 2020; Brasil et al. 2021). Our results indicate that all tested CFSs exert a positive modulation on NRF2 antioxidant pathway by increasing NRF2 nuclear activation, both under basal condition and in LPS-treated cells, with particularly relevant effects by specific probiotics, i.e., *L. plantarum* Lp10 and *L. rhamnosus* Lrh19. These findings are in line with recently published data, demonstrating that *L. plantarum* CFS improved the antioxidant capacity of the hippocampus by increasing NRF2 and SOD2 levels in an animal model of cognitive dysfunction (Xu et al. 2022).

NRF2 is a key regulator of the cellular response to inflammatory and oxidative stress (Huang et al. 2015; Tonelli et al. 2018). The protective role of NRF2 pathway seems particularly relevant in the context of neuronal damage, and has potential for the clinical management of neurodegenerative and neuroinflammation-associated diseases (Nakano-Kobayashi et al. 2020; Qu et al. 2020). Studies revealed that NRF2 knockout mice had neurological and cognitive deficiencies (Branca et al. 2017; Sigfridsson et al. 2020). In accordance with the observed increase in NRF2 activity in the nuclear fractions, gene expression analyses, in our study, revealed an up-regulation of several antioxidant enzymes controlled by NRF2, in CFSs-treated microglia, with or without LPS. Interestingly, LPS alone induces a slight nuclear increase of NRF2 activity which, in turn, should trigger the transcriptional levels of some antioxidant enzymes in a sort of cytoprotective mechanism (Chang et al. 2001; Qin et al. 2004; Pawate et al. 2004). Notably, it was observed that LPS treatment of murine BV2 microglia cells also activates NRF2 pathway and antioxidant enzymes (Li et al. 2015; Barber et al. 2023). Here, we found that treatments of LPS-activated microglia with CFSs leads to a further increase of nuclear NRF2 protein level and up-regulation of antioxidant enzymes mRNAs compared to LPS alone: this could enhance the overall ROS-scavenging capacity of the cell, thus entailing protective properties of the tested CFSs.

We found that CFSs increased SOD1 activity in microglia, thus further supporting their antioxidant and protective effects. SOD1 plays a critical role in neuroprotection (Polazzi et al. 2013). This enzyme is particularly studied in relation to brain pathophysiology. In fact, its genetic mutations are known to cause many familial forms of neurodegenerative diseases (Valentine et al. 2005; Moezzi et al. 2022). Both SOD1 and SOD2 are induced in the CNS, under inflammatory conditions (Ishihara et al. 2015; Polazzi et al. 2013; Barber et al. 2023). Previously, it was reported that activated microglia strongly express SOD2 (Ishihara et al. 2015). Intriguingly, we found that even SOD2 activity increased upon CFSs treatment (Supplementary Fig. 1), hence further corroborating the protective effects of the tested postbiotics and Lr13 particularly.

Besides activating antioxidant response, NRF2 also promotes anti-inflammatory pathways (Nakano-Kobayashi et al. 2020; Chen et al. 2006; Xu et al. 2015; Li et al. 2015). In fact, NRF2 nuclear translocation inhibits the redox-sensitive NF-kB pathway, preventing TNF-α synthesis (Huang et al. 2015; Cuadrado et al. 2014; Jin et al. 2008). TNF-α is a pleiotropic cytokine with a key role in the pathogenesis of neurodegenerative diseases (Sriram et al. 2006; Amor et al. 2010; Vincenzi et al. 2021); hence, its modulation could be critical for therapeutic purposes (Frankola et al. 2011; Amin et al. 2022). Moreover, this mediator is associated to NRF2 signaling by an autocrine loop (Rushworth et al. 2011). Earlier studies demonstrated that CFSs from lactobacilli down-regulate pro-inflammatory cytokines such as TNF-α in vivo and in vitro (De Marco et al. 2018; Cristofori et al. 2021; Peña and Versalovic 2003). In the present study, we found that pre-treatment of microglia with CFSs reversed the production of pro-inflammatory TNF-α (significantly for CFSs from Lp10 and Lrh19), while increasing the secretion of anti-inflammatory IL-10, especially when the inflammatory stimulus was added. Interestingly, it was found that the release of IL-10 from microglia induced the synaptic formation in early brain development (Lim et al. 2013); thus, probiotic CFSs could be applied in this direction by future in vitro study on neuronal cells. The pattern of expression we observed for other pro-inflammatory cytokines (i.e., IL-1β and IL-8) suggests that one of the investigated strain (Lr13) can have an overall anti-inflammatory action. These findings point to the possible use of a mixture of CFSs from the different strains to contrast microglia inflammation by their synergistic effect on cytokines modulation.

To our knowledge, this is the first attempt to investigate the anti-inflammatory and antioxidant effect of lactobacilli CFSs on human microglia cells. Just recently, bacterial conditioned media (BCM, i.e., equivalent to CFSs) from lactobacilli were tested on murine microglia cells and found to down-regulate oxidative stress and inflammation in vitro (Bulacios et al. 2023).

In conclusion, our data suggest that lactobacilli CFSs could act at the brain level to prevent and protect microglial cells from inflammation and oxidative stress, through a positive modulation of the NRF2 pathway. These results corroborate the interplay between gut and brain and the importance of this crosstalk in neurodegenerative disease. These results may represent a basis for translational studies on human brain, using the CSFs directly in ex vivo biopsies from human brain surgeries (Milior et al. 2020) and evaluating their effect on microglia–neuron interactions. Moreover, analyzing human brain and feces samples, it could be possible to link the differences in the composition and metabolites of the intestinal microbiota in patients suffering from neurodegenerative diseases, often associated with neuroinflammation. This will confirm the possibility that microbiota mediators can influence the physio-pathology of the human brain, opening to new therapeutic strategies based on postbiotics administrations.

## Supplementary Information

Below is the link to the electronic supplementary material.

Supplementary file1 (DOCX 354 kb)

## Data Availability

No datasets were generated or analysed during the current study.
