# Supplementary material for: Lactobacilli Cell-Free Supernatants Modulate Inflammation and Oxidative Stress in Human Microglia via NRF2-SOD1 Signaling
Source: Cell Mol Neurobiol. 2024 Sep 17;44:60. doi: 10.1007/s10571-024-01494-1 (PMC11408562; doi:10.1007/s10571-024-01494-1)
Supplement: Supplementary file 1 — Supplementary file1 (DOCX 354 kb) [file 10571_2024_1494_MOESM1_ESM.docx]

**Lactobacilli cell free supernatants modulate inflammation and oxidative stress in human microglia via NRF2-SOD1 signalling**

*Cellular and Molecular Neurobiology*

Mariagiovanna Di Chiano^1§^, Maria Teresa Rocchetti^2§^, Giuseppe Spano^3^, Pasquale Russo^4^, Caterina Allegretta^5^, Giampaolo Milior^6^, Raffaella Maria Gadaleta^7^, Fabio Sallustio^8^, Paola Pontrelli^8^, Loreto Gesualdo^8^, Carlo Avolio^5^, Daniela Fiocco^2^*, Anna Gallone^1^

**Affiliations**

^1^Department of Translational Biomedicine and Neuroscience (DiBraiN), University of Bari Aldo Moro, Bari, Italy

^2^ Department of Clinical and Experimental Medicine, University of Foggia, Foggia, Italy

^3^Department of Agriculture Food Natural Science Engineering (DAFNE), University of Foggia, Foggia, Italy

^4^Department of Food, Environmental and Nutritional Sciences, University of Milan, Milan, Italy

^5^Department of Medical and Surgical Sciences, University of Foggia, Foggia, Italy

^6^CIRB, Collège de France, Université PSL, CNRS, INSERM, 75005 Paris, France

^7^Department of Interdisciplinary Medicine (DIM), University of Bari Aldo Moro, Bari, Italy

^8^Department of Precision and Regenerative Medicine and Ionian Area (DiMePRe-J), University of Bari Aldo Moro, Bari Italy

^§^equal contribution

*corresponding author: [daniela.fiocco@unifg.it](mailto:daniela.fiocco@unifg.it)

**TABLE S1.** Cytotoxicity tests. Effects of different percentages (10 % and 5 % (v/v)) of CFS on viability of HMC3 cells. Cells were cultured in presence of lactobacilli-derived CFS or unconditioned medium (MRS) for 24 h. Relative cell viability (%) = [(OD595 sample – OD595 blank)/ (OD595 control – OD595 blank)] x 100. Mean and SD from at least 2 experiments performed in triplicates**.**

| ***Conditions (v/v)*** | ***Cell viability (%)*** |
| --- | --- |
| *MRS 5%* | *86.4 ± 13.3* |
| *MRS 10%* | *85.1 ± 12.6* |
| ***Lrh19 CFS 5%*** | ***90.9 ± 15.4*** |
| *Lrh19 CFS 10%* | *78.3 ± 2.5* |
| ***Lp10 CFS 5%*** | ***81.8 ± 3.1*** |
| *Lp10 CFS 10%* | *64.4 ± 34.5* |
| ***Lr 13 CFS 5%*** | ***80.0 ± 2.7*** |
| *Lr13 CFS 10%* | *89.7 ± 3.8* |

**TABLE S2.** Oligonucleotide primers utilized in real-time RT-PCR.

| **primer name** | **Nucleotide Sequences (5’🡪3’)** |
| --- | --- |
| GAPDH Fw | CGACCACTTTGTCAAGCTCA |
| GAPDH Rv | AGGGGTCTACATGGCAACTG |
| β-actin fw | CACCATTGGCAATGAGCGGTTC |
| β-actin rv | AGGTCTTTGCGGATGTCCACGT |
| SOD1 fw | CTCACTCTCAGGAGACCATTGC |
| SOD1 rv | CCACAAGCCAAACGACTTCCAG |
| CAT fw | GTGCGGAGATTCAACACTGCCA |
| CAT rv | CGGCAATGTTCTCACACAGACG |
| GST fw | ATTAACCCTCACTAAAGGGAGATATGGTGAAGGAATGATGGGGT |
| GST rv | TAATACGACTCACTATAGGGGGATCTTGGGCCGGGCACTGA |
| GPx fw | GTGCTCGGCTTCCCGTGCAAC |
| GPx rv | CTCGAAGAGCATGAAGTTGGGC |
| HO-1 fw | CCAGGCAGAGAATGCTGAGTTC |
| HO-1 rv | AAGACTGGGCTCTCCTTGTTGC |
| IL-1β F | ATGATGGCTTATTACAGTGGCAA |
| IL-1β R | GTCGGAGATTCGTAGCTGGA |

**
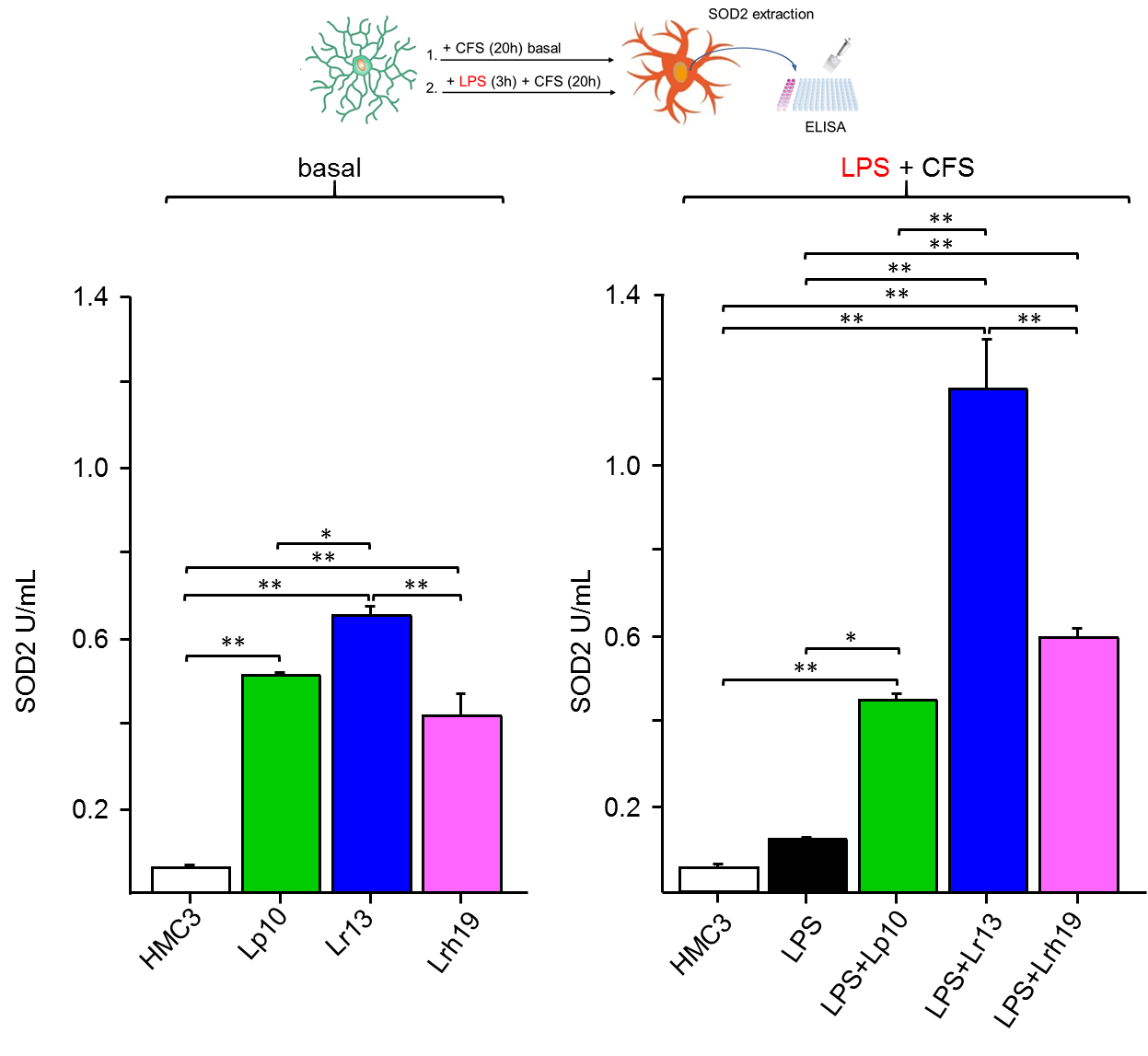
**

**Fig. S1** Activity of SOD2. SOD2 activity levels were assessed by the colorimetric activity assay in untreated microglia (open bar), in LPS-treated cells (solid bar), and in cells treated for 20 h with CFS (5%, v/v) from each bacterial strain (Lp10, Lr13, Lhr19) without or following 3 h LPS (1 μg/mL) stimulation. The results represent the mean from three independent experiments; statistically significant differences between groups were determined by one way ANOVA and post hoc Tukey test. * *p* < 0.05; ** *p* < 0.01.


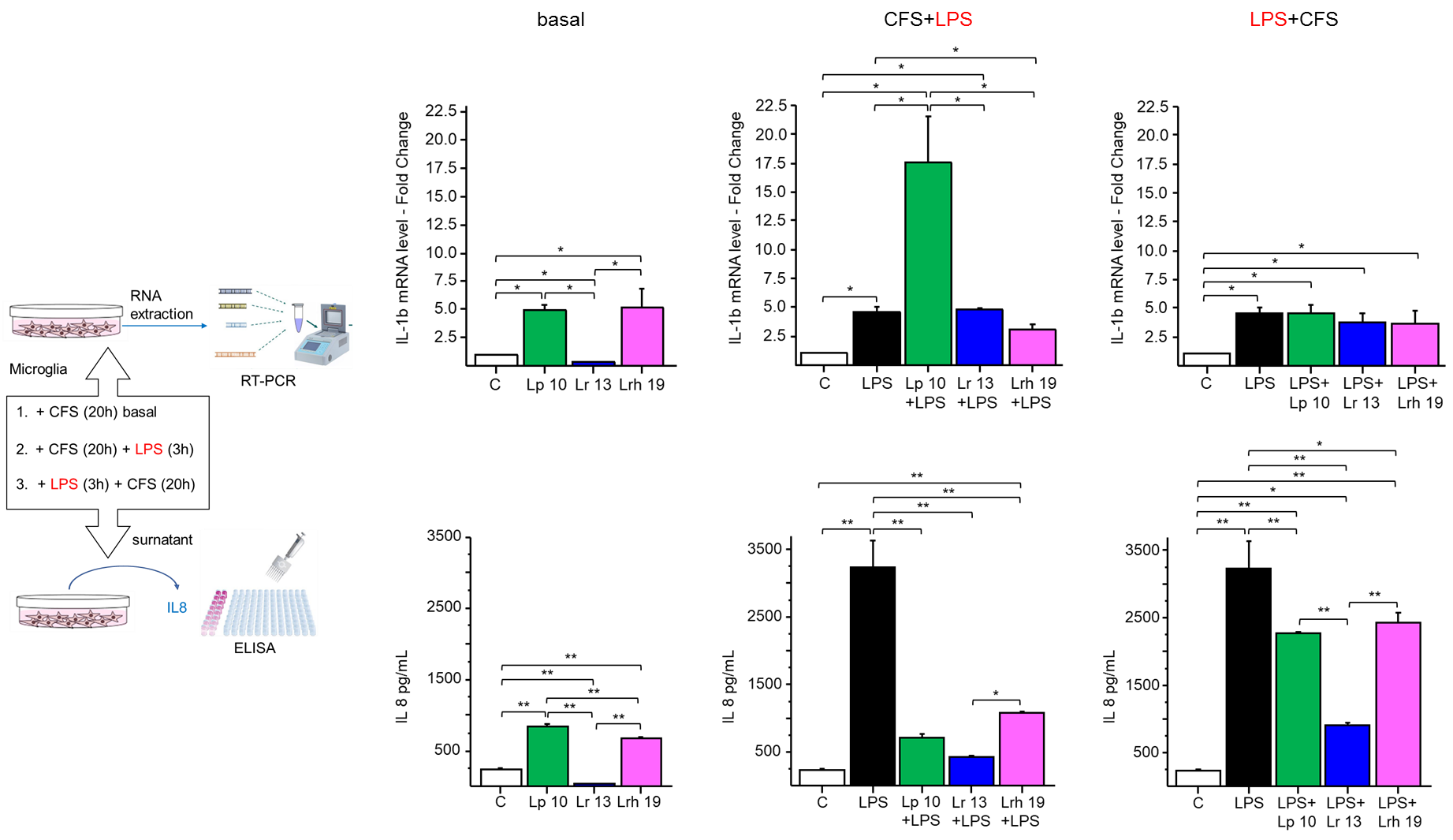


**Fig. S2 Expression of proinflammatory cytokines.** The transcriptional level of IL-1β gene (upper panel) was determined by quantitative RT-PCR. Relative mRNA levels were obtained by normalizing to those of untreated control cells (C). β-actin and glyceraldehyde-3-phosphate dehydrogenase (GAPDH) were used as housekeeping genes to normalize the expression by the ΔΔCt method. The concentration (pg/ml) of secreted IL-8 (lower panel) was determined by ELISA. Both transcriptional analysis and ELISA were performed on untreated microglia (control, white bars) and in microglia treated for 20 h with CFS from the different bacterial species (basal); in 3 h LPS-treated microglia (solid bars) and in 3 h LPS-treated microglia pre-incubated with CFS for 20 h (CFS + LPS); in 3 h LPS-treated microglia (solid bars) and in 20 h CFS-incubated microglia following 3 h LPS stimulation (LPS + CFS). Mean and SD from three independent experiments. Statistically significant differences were determined by Mann-Whitney U test or by one-way ANOVA with Tukey's multiple comparisons test, as appropriate (* *p*<0.05; ** *p*<0.01).
